# P-1079. Evaluation of cefepime/zidebactam (FPZ) dynamics in vitro using clinical Pseudomonas aeruginosa strains obtained from a patient treated with FPZ

**DOI:** 10.1093/ofid/ofaf695.1274

**Published:** 2026-01-11

**Authors:** Valliammai Alaguvel, Ayesha Khan, Erlinda R Ulloa

**Affiliations:** University of California, Irvine, California; University of California-Irvine Health; University of California Irvine School of Medicine, Irvine, California

## Abstract

**Background:**

Multidrug-resistant *P. aeruginosa* infections are a major global public health threat, often leaving clinicians with few to no treatment options. Here, we evaluated the activity of cefepime-zidebactam (FPZ), a novel β-lactam/β-lactam-enhancer agent, against 8 metallo-β-lactamase (NDM) producing *P. aeruginosa* strains longitudinally isolated from a patient treated with FPZ under compassionate use.

In 2024, the Clinical Laboratory and Standards Institute set an investigational, susceptible FPZ MIC breakpoint of ≤64µg/ml for *P. aeruginosa, Acinetobacter* and Enterobacterales. However, an FPZ MIC is only a proxy of the cefepime minimum elongation concentration (MEC) and zidebactam minimum spheroplast-forming concentration (MSC). Rather than FPZ MICs, MSCs and MECs drive pharmacokinetic/ pharmacodynamic (PK/PD) parameters. Additionally, there is a lack of *in vitro, in vivo* and clinical data on *P. aeruginosa* isolates with FPZ MICs >64.

Here, we tested 8 serial patient isolates showing a progressive increase in FPZ MICs on treatment. We aimed to assess correlation between FPZ pharmacodynamics and MIC values generated by reference antimicrobial susceptibility testing.

**Methods:**

FPZ MICs of isolates were determined by broth microdilution per CLSI guidelines. Microscopy was used to determine cefepime MECs and zidebactam MSCs (i.e., morphological changes visualized after treating 10^6^ CFU/mL of cells with each agent for 3h).

**Results:**

The isolates, except the index strain, were non-susceptible to all FDA-approved agents, including cefiderocol, ceftolozane/tazobactam, and ceftazidime/avibactam. FPZ MICs ranged from 16 to >512 over treatment. Strains with FPZ MICs of 16-32, which were isolated before FPZ therapy, had MECs of 64-128 (4x the MIC) and MSCs of 8-16 (1/2x the MIC). Strains with MICs of 128->512, which were isolated at the end of FPZ therapy, had MECs of 128-512 (near MIC) and MSCs of 16-128 (1/8x the MIC).

**Conclusion:**

At high FPZ MICs, zidebactam MSCs remain 8-fold lower than MICs. This suggests that FPZ may retain bactericidal activity even when MICs exceed the investigational susceptibility breakpoint of 64. This study provides insight into FPZ's therapeutic potential and highlights the importance of dissecting PK/PD dynamics in strains showing evolution of high MICs.

**Disclosures:**

All Authors: No reported disclosures

